# Synchronizing Germination Rates Across Plant Species for Fabricated Ecosystems EcoFAB 2.0

**DOI:** 10.21769/BioProtoc.5537

**Published:** 2025-12-05

**Authors:** Romane S. F. Charbeaux, Vicky J. Waymouth, Jacob Calabria, Troy Miller, Peter Andeer, Michelle Watt

**Affiliations:** 1 School of Biosciences, Faculty of Science, The University of Melbourne, Melbourne, Australia; 2 Environmental Genomics and Systems Biology, Lawrence Berkeley National Laboratory, Berkeley, CA, USA; 3 School of Molecular Sciences, University of Western Australia, Crawley, Perth, Australia

**Keywords:** Fabricated ecosystem, Live root imaging, Gnotobiotic, Germination, *Arabidopsis thaliana*, *Fragaria vesca*, *Lactuca sativa*

## Abstract

Roots are essential organs for plants, facilitating water and nutrient uptake from the soil to support growth. Traditional methods for studying root systems, such as rhizoboxes and rhizotrons, have provided valuable insights. However, advanced methods such as fabricated ecosystems (EcoFAB) combined with new generation microscopes now enable a more detailed investigation of the rhizosphere, the microenvironment surrounding roots, allowing a deeper understanding of root tissue, exudates, and plant–soil interactions. This microenvironment can be used to investigate the adaptation of plants to environmental stress (salinity, drought, higher temperatures). Our procedure focuses on establishing standardized protocols for plant growth tailored to the EcoFAB system, which offers a controlled environment to study root dynamics. This work also contributes new insights into the early stages of plant germination, an area currently underexplored in the literature. While numerous studies focus on plant growth or genetic aspects, such as gene induction, the germination phase remains underexplored. We have developed optimized germination protocols for multiple plant species, ensuring uniform seedling size and sufficient development for seamless integration into the EcoFAB system.

Key features

• Optimized the germination protocol for plant rhizosphere studies using the EcoFAB 2.0 system.

• Adapted the well-established protocol for *Brachypodium distachyon* to other species, including *Arabidopsis thaliana, Fragaria vesca*, and *Lactuca sativa*.

• Preliminary measurements of root and leaf traits were taken to determine the best method.

## Graphical overview


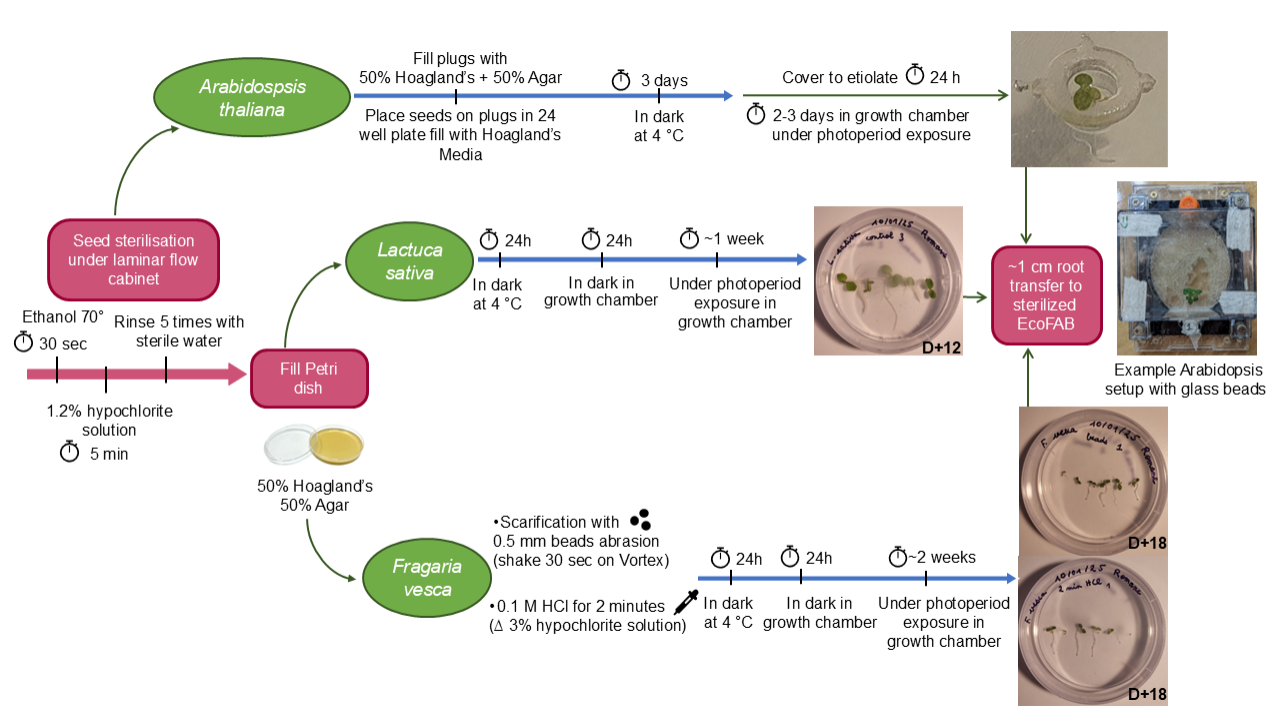


**Seed preparation procedure and initial growth prior to transfer to fabricated ecosystems EcoFAB 2.0 for *Arabidopsis thaliana, Lactuca sativa*, and *Fragaria vesca*.**

## Background

The understanding of root development and its surrounding environment, the rhizosphere, under varying conditions is crucial for optimizing plant growth and yield for agriculture. The study of plant physiological processes in response to their environment is possible with fabricated ecosystems (EcoFAB 2.0 system). This system allows high-resolution imaging of roots in a multi-factorial, controlled environment [1,2] and the characterization of shoot growth. EcoFABs enable the study of plant–microbiome interactions by introducing microbial, fungal, or bacterial cultures into the chamber, which can be easily sterilized. During experiments, the nutrient media can be replaced or altered at any time. Imaging or scanning can be conducted to collect data on root size, root hairs, hyphae, leaves, and interparticle space. Root architecture and leaf growth can be analyzed simultaneously. By imposing different conditions on the plants, we can observe which ones encourage or hinder their development.

The development of the EcoFAB system, a repeatable and versatile tool for studying plants in controlled environments, enables a more effective transfer of knowledge across different scientific communities. A standardized sterilization and germination protocol for various plant species is necessary to ensure uniform plant growth stages suitable for transfer into the sterilized EcoFAB system. Seed priming allows the control of seed hydration and induces physiological, biochemical, and molecular alterations that trigger germination [3]. With this type of treatment, it is possible to increase vigor and germination rate or reduce the time needed to obtain better seedlings. Radicle protrusion through the seed coat is the main event to observe homogeneous germination. To enhance germination, many invasive methods [4,5] have been described in the literature, mainly based on water uptake by seeds. This is why we developed a user-friendly seed treatment and germination protocol for different plant species to achieve a homogeneous germination rate and growth suitable for experiments in the EcoFAB system.

Several other tools are available to study plants and their interactions with soil, microorganisms, and ecosystems. Systems like rhizoboxes and rhizotrons [6] were initially created to study root growth in mature plants. These systems enable plants to grow in their natural soil environment. Using a non-destructive method, root or shoot growth can be assessed through successive measurements on the same plants. This approach reduces phenotypic variability linked to differences between individual plants and enhances accuracy compared to methods requiring plant destruction during sampling [7]. Recently, the CD-Rhizotron system [8] was developed to study the spatial differentiation of roots linked to different microbial communities or exudates. Fine root studies are also possible with Minirhizotrons [9] or EnRoot [10] devices, but as with previous methods, the ecosystem is not controlled, and plants remain in the field. More recent technologies, such as TRIS [11] and root arrays [12], have been developed to achieve a precise understanding of root behavior in microfluidic devices, imaging them in real-time and enabling genotyping analysis. A key limitation of these devices is their restriction to experiments on small, young plants. Additionally, these systems do not replicate field-like environments.

In contrast, EcoFABs are fully controllable hydroponics systems, offering many possibilities for experiments. A deep understanding of plant growth mechanisms and interactions has numerous direct applications in agriculture, such as improving yield and triggering some diseases caused by fungi or bacteria. Conservation efforts for endangered species are another important application, as the International Union for Conservation of Nature (IUCN) red list encourages the scientific community to engage in conservation efforts for these species. Early developmental stages are crucial for the conservation of endangered species, and EcoFABs could help determine key features of early development, particularly by optimizing life cycles and seed germination. Beyond these applications, studying plant–microbiome–soil interactions in systems like the EcoFAB holds significant potential for space exploration. As part of the Plants 4 Space Program, this research explores the use of EcoFABs in optimizing fruit and vegetable production for long-term space missions. A deep understanding of root cells and rhizosphere interactions is essential for optimizing crops in space [13], especially fresh food production to counteract the degradation of packaged food and the potential digestive issues caused by a lack of fruits and vegetables in astronauts’ diets [14]. This work introduces the potential use of strawberries [15] and lettuce in EcoFAB 2.0, aligning with space programs that have mainly focused on these crops. It also opens the door to studying additional plants in the EcoFAB system.

Inspired by the *Brachypodium distachyon* protocol [16,17], we present a precise germination and setup protocol in EcoFABs for *Arabidopsis thaliana* with the aim of extending the study of this model plant. Lettuce is widely studied in hydroponic systems for vegetable production [18,19]. Here, we developed a new approach to studying lettuce (*Lactuca sativa*; Outredgeous) in its early stages of development. We also propose a quite similar protocol applicable for *Fragaria vesca* (Hawaii 4). Our protocol can be easily adapted to many other species, including endangered ones. The main goal is to extend EcoFAB 2.0's applicability to additional plant species and provide standardized procedures that improve both the quantitative (germination rate) and qualitative (uniformity) aspects of plant germination.

## Materials and reagents

**Biological materials**

1. *Arabidopsis* seeds (Columbia, wild-type seeds from the Arabidopsis Biological Resource Center at The Ohio State University; seeds were bulked up from that stock in our lab)

2. *Lactuca sativa “Outredgeous”* seeds (The Seed collection, Z-10139_P)

3. *Fragaria vesca “Hawaii 4”* seeds (original seeds from https://npgsweb.ars-grin.gov/gringlobal/accessiondetail?id=1856850 ; seeds were bulked up from that stock in our lab)

**Reagents**

1. Plant cell culture agar powder (Sigma-Aldrich, catalog number: A1296-5KG)

2. 100% ethanol (Bio21, catalog number: EA043-2.5L-J)

3. Sodium hypochlorite solution 12% (Thermo Fisher Scientific, catalog number: AJA485-5L)

4. Triton^TM^ X-100 (Sigma-Aldrich, catalog number: X100-100ML)

5. Hydrochloric acid (HCl), 37% (Sigma-Aldrich, catalog number: 258148-500ML, CAS 7647-01-0)

6. Potassium nitrate (KNO_3_) (Sigma-Aldrich, CAS number: 7757-79-1)

7. Ammonium dihydrogen phosphate [(NH_4_)_2_HPO_4_] (Sigma-Aldrich, CAS number: 7783-28-0)

8. Calcium nitrate tetrahydrate [Ca(NO_3_)_2_·4H_2_O] (Sigma-Aldrich, CAS number: 13477-34-4)

9. Magnesium sulphate heptahydrate (MgSO_4_·7H_2_O) (Sigma-Aldrich, CAS number: 10034-99-8)

10. Boric acid (H_3_BO_3_) (Sigma-Aldrich, CAS number: 10043-35-3)

11. Manganese chloride tetrahydrate (MnCl_2_·4H_2_O) (Sigma-Aldrich, CAS number: 13446-34-9)

12. Zinc sulphate heptahydrate (ZnSO_4_·7H_2_O) (Sigma-Aldrich, CAS number: 744620-0)

13. Copper sulphate pentahydrate (CuSO_4_·5H_2_O) (Sigma-Aldrich, CAS number: 7758-99-8)

14. Sodium molybdate dihydrate (Na_2_MoO_4_·2H_2_O) (Sigma-Aldrich, CAS number: 10102-40-6)

15. Iron (II) sulphate heptahydrate (FeSO_4_·7H_2_O) (Sigma-Aldrich, CAS number: 7782-63-0)

16. EDTA disodium salt (C_10_H_14_N_2_Na_2_O_8_) (Sigma-Aldrich, CAS number: 6381-92)

**Solutions**

1. Sodium hypochlorite solution 1.2% (see Recipes)

2. Sodium hypochlorite solution 3% (see Recipes)

3. Hoagland’s nutrient solution [20]

a. Micronutrients solution (see Recipes)

b. Macronutrients solution (see Recipes)

c. Iron solution (see Recipes)

d. Final Hoagland’s nutrient solution (see Recipes)

**Recipes**

**1. Sodium hypochlorite solution 1.2%**

**Table:** 

Reagent	Final concentration	Volume
Sterile MilliQ water		43 mL
Triton X-100	0.106 g/mL	2 mL
Sodium hypochlorite solution (12%)	12 g/L	5 mL
Total		50 mL

Prepare under sterile conditions in a laminar flow cabinet to minimize contamination. We recommend storing it at 4 °C and making it fresh every fortnight.

**2. Sodium hypochlorite solution 3%**

**Table:** 

Reagent	Final concentration	Volume
Sterile MilliQ water		35.5 mL
Triton X-100	0.106 g/mL	2 mL
Sodium hypochlorite solution (12%)	37.2 g//L	15.5 mL
Total		50 mL

Prepare under sterile conditions in a laminar flow cabinet to minimize contamination. We recommend storing it at 4 °C and making it fresh every fortnight.

**3. Hoagland’s nutrient solution**

Create a collection of concentrated stock solutions for each ingredient that can be filter-sterilized. Dissolve the following salts in individual 50 mL aliquots of MilliQ water. Under a laminar flow, filter-sterilize each stock solution into autoclaved bottles. Store in the dark and at room temperature.

**a. Macronutrient solutions (four separate solutions)**

**Table:** 

Reagent	Final concentration	Quantity or volume
KNO_3_	303.4 g/L	15.17 g
NH_4_H_2_PO_4_	115 g/L	5.75 g
Ca(NO_3_)_2_·4H_2_O	944.6 g/L	47.23 g
MgSO_4_·7H_2_O	493 g/L	24.65 g

**b. Micronutrient solution**

**Table:** 

Reagent	Final concentration	Quantity or volume
H_3_BO_3_	2.86 g/L	143 mg
MnCl_2_·4H_2_O	3.76 g/L	188.1 mg
CuSO_4_·5H_2_O	0.13 g/L	6.3 mg
ZnSO_4_·7H_2_O	0.39 g/L	19.6 mg
Na_2_MoO_4_·2H_2_O	0.03 g/L	1.3 mg

**c. Iron solution**

**Table:** 

Reagent	Final concentration	Quantity or volume
Fe(III)SO_4_·7H_2_O	5.56 g/L	278 mg
C_10_H_14_N_2_Na_2_O_8_	7.44 g/L	372 mg

Make sure to minimize light exposure of the iron solution by covering the autoclaved bottles with aluminum foil.

**d. Final Hoagland’s nutrient solution**

**Table:** 

Reagent	Final concentration	Quantity or Volume
Sterile MilliQ water		1 L
KNO_3_	0.607 g/L	0.607 g
NH_4_H_2_PO_4_	0.115 g/L	0.115 g
Ca(NO_3_)_2_·4H_2_O	0.995 g/L	0.995 g
MgSO_4_·7H_2_O	0.493 g/L	0.493 g
H_3_BO_3_	2.8 mg/L	2.8 mg
MnCl_2_·4H_2_O	3.762 mg/L	3.762 mg
CuSO_4_·5H_2_O	0.392 mg/L	0.392 mg
ZnSO_4_·7H_2_O	0.126 mg/L	0.126 mg
Na_2_MoO_4_·2H_2_O	0.026 mg/L	0.026 mg
Fe(III)SO_4_·7H_2_O	5.56 mg/L	5.56 mg
C_10_H_14_N_2_Na_2_O_8_	7.44 mg/L	7.44 mg
Total		1 L

Hoagland’s nutrient solution is prepared by adding 2 mL of KNO_3_ and 1 mL of all other stock solutions [NH_4_H_2_PO_4_, Ca(NO_3_)_2_·4H_2_O, MgSO_4_·7H_2_O, micronutrient solution, and iron solution] to 991 mL of MilliQ water in an autoclaved bottle under sterile conditions in a laminar flow cabinet. The pH is adjusted to 6 mainly so that plants and microorganisms can also grow in this media. This yields a sterile Hoagland’s nutrient solution with the composition described above. Store it at room temperature.

**Laboratory supplies**

1. 24-well tissue culture treated plates, with lid, flat bottom, sterile (Adelab Scientific, catalog number: CNG3526; referred to as 24-well plates)

2. Petri dish 90 × 25 mm (Thermo Fisher Scientific, catalog number: LBS60014X)

3. Petri dish 90 × 15 mm (Thermo Fisher Scientific, catalog number: LBS60001)

4. Plugs, made by Lawrence Berkeley National Laboratory in collaboration with us; size-V3 plugs worked best (more information can be found at https://eco-fab.org/)

5. Pipette tips 100–1,000 μL (Mettler-Toledo, catalog number: 17014969)

6. Pipette tips 100–200 μL (Mettler-Toledo, catalog number: 17014964)

7. Falcon^®^ 50 mL, conical bottom, sterile (Bio-Strategy Pty Limited, catalog number: 352070)

8. Eppendorf tube 2 mL (Eppendorf, catalog number: 0030120094)

9. Borosilicate beads 0.5 mm diameter (Merck, catalog number: Z250465)

10. Forcep jeweller (McFarlane Medical, catalog number: 34288-LQ)

11. Micropore tape (3M, catalog number: 1530-0)

12. Aluminum foil

13. EcoFAB 2.0 (more information can be found at https://eco-fab.org/)

14. Screwdriver (Panasonic, EY 7412 SB, Cordless Screw Driver)

## Equipment

1. Laminar flow cabinet (Clyde-Apac, model: HWS120)

2. Vortex mixer (Ratek, model: VM1)

3. Growth chamber (Bioline Global, model: AR-22L)

4. Freezer

5. Autoclave

6. EVOS Microscope (Thermo Fisher Scientific, model: EVOS M7000 Imaging System)

7. WinRHIZO Scanning system (Regent Instruments)

## Software and datasets

1. Celleste^TM^ 5 Image Analysis Software (Thermo Fisher Scientific, AMEP4877) or Fiji image analysis, free software package available on GitHub

## Procedure

This protocol is based on the well-known protocol developed for *Brachypodium distachyon* [2]. We provide the optimal procedure identified after testing multiple alternatives. The supplementary data includes the alternative methods tested, their results, and the conclusions drawn for each. Note that every batch of seed has its own germination rate. For each step, ensure that all equipment is sterile and placed inside a laminar flow cabinet.

**A. *Arabidopsis***

1. Prepare the plate and plugs

a. All solutions and plugs should be sterilized and autoclaved before starting this step.

b. In the laminar flow hood, carefully mix Hoagland’s nutrient solution and hot agar as indicated in Table 1.

**Table 1.** Germination medium for *Arabidopsis*

Reagent	Final concentration	Volume
Hoagland’s 1× concentration nutrient solution		25 mL
Agar 1.3%	6.5 g/L	25 mL
Total	n/a	50 mL

c. Place plugs upright in 24-well plates and pipette a drop of the prepared germination medium for *Arabidopsis* into each plug (around 5 μL).

d. Move to the side of the laminar flow cabinet to cool.

2. Seed sterilization

a. Aliquot a small number of seeds in an Eppendorf tube.

b. Add 1 mL of 70% ethanol and gently shake for 30 s.

c. Decant and remove all ethanol using a pipette.

d. Transfer seeds to 1.2% sodium hypochlorite solution, leave, and gently shake for 5 min.

e. Decant the solution.

f. Rinse seeds five times with autoclaved Milli-Q water.

3. Seed setup

a. Check if the medium has solidified into the plug.

b. Pipette 1 mL of Hoagland’s nutrient solution into each well of the 24-well plate. Be gentle to ensure the plugs stay upright.

c. Place the sterilized seeds directly on plugs using a pipette. Since the seeds are small, they can be suspended in a small amount of sterile Milli-Q water. Unlock the pipette and slowly release the solution, aiming to deposit ideally two seeds per plug.

d. Put the lid and seal the plate with micropore tape.

e. Wrap the plate in aluminum foil and place it in a dark, cold room (3 days at 4 °C) for cold stratification.

4. Induction of shoot development

a. Move plates to the growth cabinet, keeping them covered in aluminum foil (2–3 days at 20 °C).

b. Remove the foil and keep in the growth chamber under your defined photoperiod (14 h light 26 °C/10 h dark 20 °C, 110–140 μmole·s^-1^·m^-2^).

**Figure 1.**
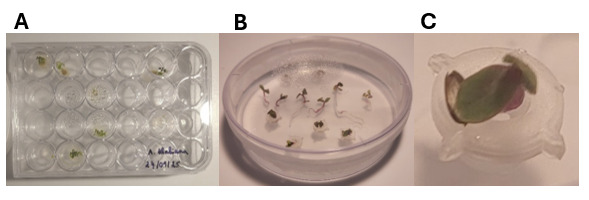
Experimental setup of *Arabidopsis* seeds for germination growth assays in EcoFAB 2.0. (A) Setup of seed in 24-well plates to test different methods. (B) Single seeds on agar without a plug or in a plug, ready for setup in EcoFAB 2.0. (C) Single seed on agar in a plug, ready for setup in EcoFAB 2.0.

**Figure 2.**
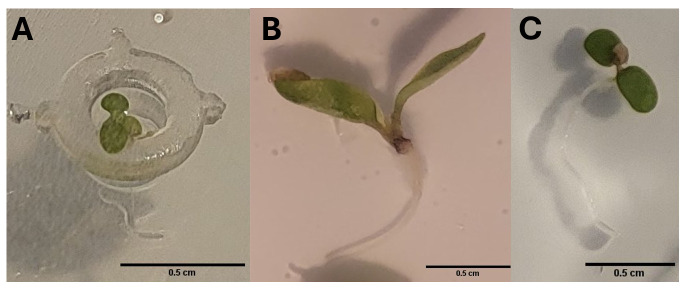
Images of the optimal seedling stage for transfer into EcoFABs for each plant. (A) *A. thaliana.* (B) *Lactuca sativa.* (C) *Fragaria vesca.*

5. Seedling transfer to EcoFAB 2.0: Wait up to 5 days, depending on plant growth. Plants are ready to set up into EcoFAB when we can see leaves and small roots (example in Figures 1C and 2A).

6. Begin further interventions, which may include live imaging, exudate collection, introduction of microorganisms, and genetic analysis.

**B. *Lactuca sativa* “Outredgeous”**

1. Prepare the Petri dish

a. In the laminar flow hood, carefully mix Hoagland’s nutrient solution and hot agar in a Falcon tube, using the quantities indicated in Table 2.

**Table 2.** Germination medium for *Lactuca sativa*

Reagent	Final concentration	Volume
Hoagland’s 1× concentration nutrient solution		25 mL
Agar 1.5%	7.5 g/L	25 mL
Total	n/a	50 mL

b. Fill each large Petri dish with approximately 20 mL of medium.

c. Move to the side of the laminar flow cabinet to cool.

2. Seed sterilization

a. Aliquot a small number of seeds in an Eppendorf tube.

b. Add 1 mL of 70% ethanol and gently shake for 30 s.

c. Decant and remove all ethanol using a pipette.

d. Transfer seeds to 1.2% sodium hypochlorite solution, leave them, and gently shake for 5 min.

e. Decant the solution.

f. Rinse seeds five times with autoclaved Milli-Q water.

3. Seed setup

a. Using sterile forceps, place the sterile seeds directly on the medium with the same side facing upward toward the lid and the same orientation.

b. Put the lid and seal the plate with micropore tape.

c. Wrap the plate in aluminum foil and place it in a dark, cold room (1 day at 4 °C) for cold stratification.

4. Induction of shoot development

a. Remove the foil and move the plates to a growth cabinet under a defined photoperiod (14 h light 26 °C/10 h dark 20 °C, 110–140 μmoles·s^-1^·m^-2^).

b. Wait a further day to tilt the plate: incline the Petri dish by elevating the top edge by 3 cm to create a slight tilt directing roots to grow downward. Keep in the growth chamber.

5. Seedlings transfer to EcoFAB 2.0: Wait for radicle protrusion (approximately 1 week), defined as the emergence of the root tip from the seed. At the protrusion stage, a small root is visible (as shown in the seedling on the right side of Figure 2B). Plants are ready to be set up in EcoFAB when we can see leaves and roots (approximately 1–1.5 cm in length).

6. Begin further interventions, which may include live imaging, exudate collection, introduction of microorganisms, and genetic analysis.

**C. *Fragaria vesca* “Hawaii 4”**

1. Prepare the Petri dish

a. In a sterile laminar flow hood, carefully mix Hoagland’s nutrient solution and hot agar in a Falcon tube, using the quantities indicated in Table 3.

**Table 3.** Germination medium for *Fragaria vesca*

Reagent	Final concentration	Volume
Hoagland’s 1× concentration nutrient solution		25 mL
Agar 1.4%	7 g/L	25 mL
Total	n/a	50 L

b. Fill each large Petri dish with approximately 20 mL of medium.

c. Move to the side of the laminar flow cabinet to cool.

2. Seed priming treatment

a. Aliquot a small number of seeds in an Eppendorf tube.

b. Scarification process: Add 20 sterilized 0.5 mm borosilicate beads to the Eppendorf tube. Shake for 30 s using the vortex mixer to create small abrasions on the seed coat.

*Note: See Problem 1 in Troubleshooting.*

3. Seed sterilization

a. Aliquot a small number of seeds in an Eppendorf tube.

b. Add 1 mL of 70% ethanol and gently shake for 30 s.

c. Decant and remove all ethanol using a pipette.

d. Transfer seeds to 3% sodium hypochlorite solution (see note), then leave and gently shake for 5 min.

e. Decant the solution.

f. Rinse seeds five times with autoclaved Milli-Q water.

*Note: See Problem 2 in Troubleshooting.*

4. Seed setup

a. Using forceps, place the sterile seeds directly on the medium with the same orientation.

b. Put the lid and seal the plate with micropore tape.

5. Induction of the end of dormancy: Wrap the plate in aluminum foil and place it in a dark, cold room (1 day at 4 °C) for cold stratification.

6. Induction of shoot development

a. Remove the foil and move the plates to a growth cabinet under a defined photoperiod (14 h light 26 °C/10 h dark 20 °C, 110–140 µmoles·s^-1^·m^-2^).

b. Wait a further day to tilt the plate: incline the Petri dish by elevating the top edge by 3 cm to create a slight tilt. Keep in the growth chamber.

7. Seedlings transfer to EcoFAB 2.0: Wait for radical protrusion (approximately 2 weeks), defined as the emergence of the root tip from the seed. At the protrusion stage, a small root is visible. Plants are ready to be set up in EcoFAB when we can see leaves and roots (approximately 1–1.5 cm in length).

8. Begin further interventions, which may include live imaging, exudate collection, introduction of microorganisms, and genetic analysis

**D. Seedling transfer to EcoFAB 2.0**

When plants reach the appropriate size (1 cm root length is optimal), gently insert the root into the EcoFAB plant reservoir with sterile forceps. Handle the roots carefully to avoid any damage. Examples of the optimal stage for transfer for each plant are shown in Figure 2. Be careful when watering the plant to avoid disturbing the setup.

**E. Imaging is ready**

**Figure 3.**
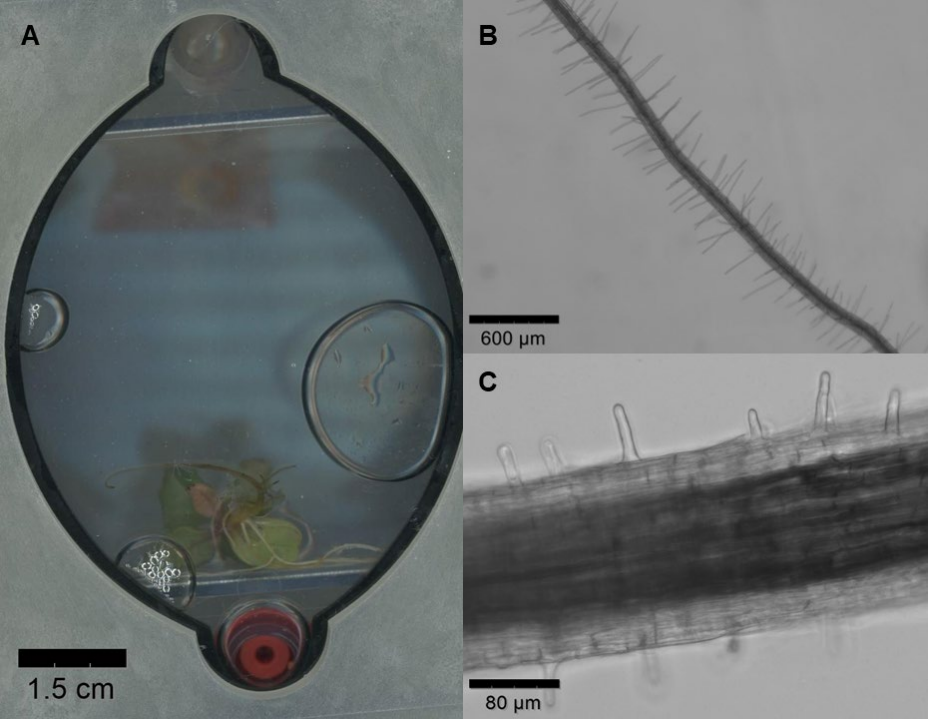
Potential imaging strategies for root systems using EcoFAB 2.0. (A) Scan of *Lactuca sativa* root system (using a Canon, Epson Expression 11000XL) under control conditions, 34 days after seed sterilization and 22 days after being placed in EcoFAB 2.0. (B) *Arabidopsis* root imaged with EVOS M7000 microscope at 4× magnification, 21 days after seed sterilization. Seeds were placed on an agar-filled plug on an agar Petri dish 14 days after transfer to EcoFAB 2.0. (C) *Fragaria vesca* root hairs imaged with EVOS M7000 microscope at 20× magnification, 34 days after seed priming with 2 min 0.1 M HCl treatment followed by sterilization.

1. Initial whole-plant images can be taken; we used a smartphone.

2. Continue to care for plants by refilling media every two days and applying the conditions specific to your study.

3. Take additional images as needed for your study (examples of microscope images in Figure 3).

## Data analysis

Whole-plant images were taken using a Samsung S21 smartphone (AMOLED 2X, 120 Hz, HDR10+ display at 403 ppi, 1080 × 2340 pixels resolution, 19.5:9 aspect ratio) from a height of 20 cm above the desk to maintain consistency. Seeds were first subjected to different seed treatment conditions prior to germination. Germination rate and germination score were recorded for each condition for *Lactuca sativa* and *Fragaria vesca*. Early developmental stages of seedlings in EcoFAB systems were monitored to ensure survival and acclimation.

Plant traits, including root length and leaf area, were quantified from images if visible using measurement tools in Celleste software, although similar analyses can also be performed with Fiji. More precise measurements of roots could be obtained using a microscope or WinRHIZO scanner (see example images in Figure 2). Measurements were taken over time to track the progression of seedling development.

Non-parametric Mann–Whitney tests were used since data did not meet the assumptions of normality. Statistical analyses were conducted in R (version 4.5.1, base program), with seed treatment and time included as fixed factors in the models (results shown in Table S1). To test for differences in the spread of data for each variable by treatment, we used Levene’s test (results shown in Table S2).

## Validation of protocol

For each plant, we tested different treatments to get homogeneous seedlings ready for EcoFAB setup. We applied different conditions to seeds that can improve germination.

**A. *Arabidopsis*B**

*Arabidopsis* seeds were too small to sit on the pore in EcoFab 2.0; they needed an additional surface (agar) and a plug to hold them and maintain the habitat of the plant. Initially, we used Murashige and Skoog medium but found poor results. So, we moved to Hoagland’s nutrient solution as recommended by Delden and colleagues [20]. We tried the following treatments using Hoagland’s nutrient solution to improve homogeneity and the rate of germination for *Arabidopsis*:

1. Seeds placed on plugs filled with growth medium and positioned on a 90 × 25 mm Petri dish.

2. Seeds placed on plugs filled with growth medium and positioned in wells of a 24-well plate filled with Hoagland’s nutrient solution.

3. Seeds placed on plugs filled with rockwool and positioned on a 90 × 25 mm Petri dish.

4. Seeds placed on plugs filled with rockwool and positioned in wells of a 24-well plate filled with Hoagland’s nutrient solution.

Germination of seeds was very high for all treatments, ranging from 86.4%–93.3%. The lowest germination rates were observed on Petri dishes, where both treatments had a germination rate of 86.4%. Germination was not scored for *Arabidopsis* as roots were not visible on rockwool.

**Figure 4.**
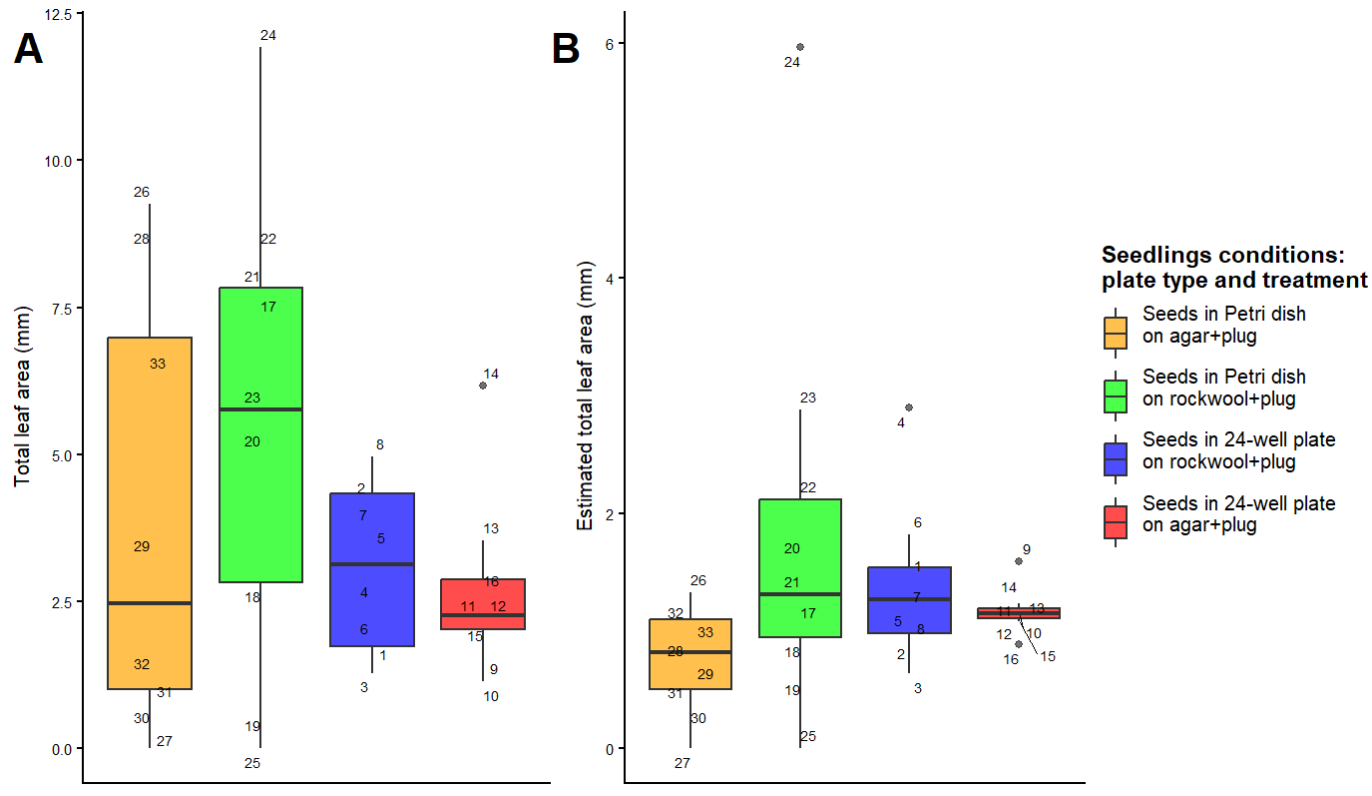
Total leaf area (A) and estimated leaf area (B) between measurements of *Arabidopsis thaliana*. One Petri dish per treatment; all treatments fit in one 24-well plate, with 8 plugs each. Each point is a number indicating individual plants.

We found no significant differences between treatments for total leaf area and our estimated leaf area per plant (leaf area/total number of plants; Figure 4 and Table S1). There were differences in the spread of data, where total leaf area (p-value = 0.03) and estimated leaf area (p-value = 0.07) of plants on 24-well plates with agar plugs were more homogeneous compared to those in other treatments (Figure 4 and Table S2). For total leaf area, there was also a significant difference with plate type, where plants grown on Petri dishes had greater variation in leaf area per plug than those grown on a 24-well plate (p-value < 0.01; Figure 4A and Table S2); also, 34% of variance in total leaf area was explained by plate type (Figure 4A and Table S2).

In addition to quantitative results, we also observed that 37.5%–44% of seedlings on plugs with rockwool had symptoms of waterlogging, primarily yellow or wilting leaves, compared to only 12.5% on agar-filled plugs.

The best treatment was an agar-filled plug in a 24-well plate with liquid Hoagland's nutrient solution. In that treatment, we observed that there were three times fewer symptoms of waterlogging. Plants from this treatment also grew more homogenously. Examples of plants that were ready to transfer to EcoFABs can be found in Figure 2A.

**B. *Lactuca sativa* “Outredgeous”**

**Figure 5.**
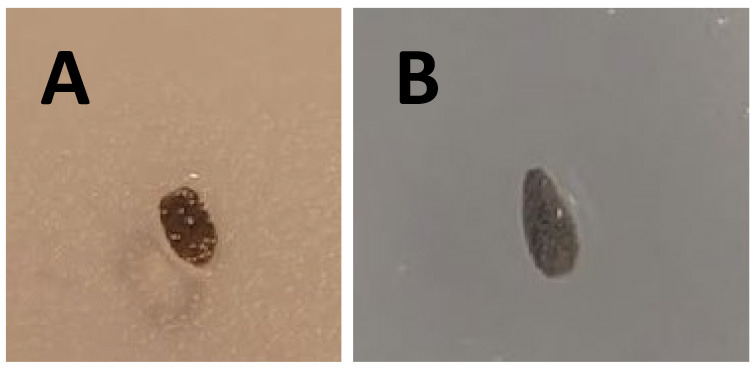
Top seed of *Lactuca sativa* cut (A) in comparison to a natural seed (B) for the control

We tried five treatments to improve homogeneity and rate of germination for *Lactuca sativa.* All treatments were applied before sterilization, except treatment 2:

1. Control (only sterilization).

2. Seeds placed on plugs (we were inspired by the setup for *Arabidopsis*, except seeds had to be placed on plugs using forceps).

3. The bottom of the seeds was carefully cut using a razor blade. This was a tricky process, as it is quite difficult to determine and control exactly where to cut (Figure 5).

4. Seeds put in darkness for 90 min in the cold room, followed by 30 min of red-light exposure in the growth chamber.

5. The bottom of the seeds was carefully cut using a razor blade; then, seeds were put in darkness for 90 min in the cold room, followed by 30 min of red-light exposure in the growth chamber.

Treating seeds with NaCl was omitted from the study due to previously reported worsened physiological reactions of *L. sativa* in the photosystem III and altered ionic balance, which resulted in a significantly lower yield of the plants [21]. In addition to this, the use of plugs (Figure 2) was not effective for *L. sativa* as the main root seems to be too large to sit well on a plug, making the pore too small and limiting plant growth.

**Figure 6.**
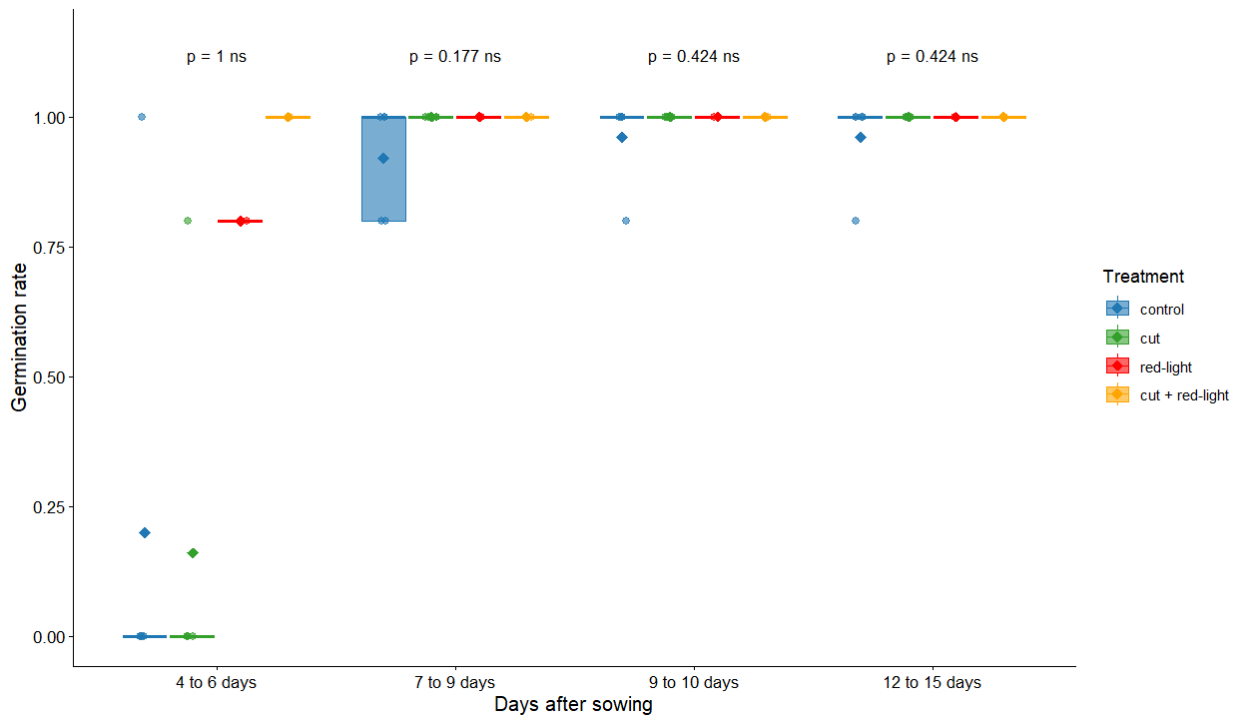
Germination rate across several seed priming treatments on *Lactuca sativa*. Points relate to the germination rate for each Petri dish. Mann–Whitney test results are displayed above the boxplots (ns: non-significant). Germination was recorded over four time intervals (4–6, 7–9, 9–10, and 12–15 days after sowing) under four treatments: control and cut (5 Petri dishes each, 6 seeds per dish), and red-light and cut plus red-light (1 Petri dish each, 6 seeds per dish).

**Figure 7.**
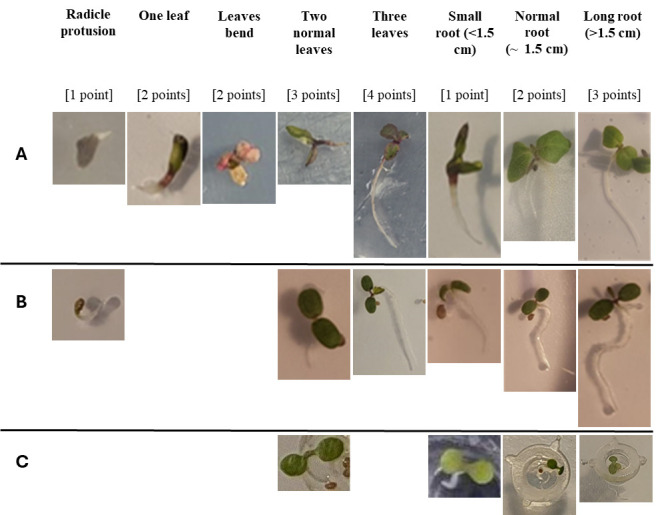
Examples of plant conditions for each germination score for *Lactuca sativa* (A) and *Fragaria vesca* (B). Scores were not used for *Arabidopsis* due to roots not being visible in rockwool, but we have placed examples of plants at different stages (C). A combination of leaf and root length scores was given to all plants based on the features that were present at the time of imaging.

We found no significant differences between treatments in germination rate (Figure 6 and Table S1). Moreover, germination rate does not fully reflect the “health” of the plant; as such, we looked at qualitative aspects of germination. Our qualitative results show that the control treatment seems to be the best way to obtain homogeneous and healthy seedlings, while red-light exposure tends to lead to plants with bent or malformed leaves instead of “normal” ones. In addition, no root growth was observed with the red-light exposure treatment. Contrary to another study [21], we did not find a response from UV radiation on seed germination. For treatment 5, which combines two factors (cut and red-light exposure) that did not improve germination quality, we did not observe any positive synergy. We therefore found it more useful to calculate the germination score based on the visual condition of the plants (see Figure 7 for examples of plants and their related germination score).

**Figure 8.**
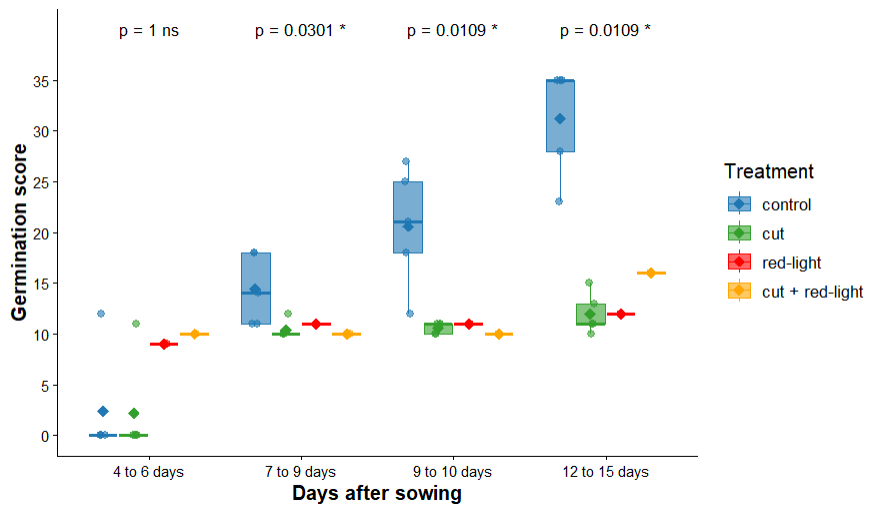
Germination score across four seed priming treatments on *Lactuca sativa*. Mann–Whitney test results are displayed above the boxplots (*p-value < 0.05, ns: non-significant). One Petri dish per time interval, 6 seeds each dish.

Germination scores were assessed over four time intervals (4–6, 7–9, 9–10, and 12–15 days after sowing) under four treatments: control and cut (5 Petri dishes each, 6 seeds per dish), and red light and cut + red light (1 Petri dish each, 6 seeds per dish). Control and cut treatments were significantly higher in control than cut treatments at 7–9, 9–10, and 12–15 days (p-value < 0.05), while no significant differences were found at 4–6 days (p = 1, ns; Figure 8 and Table S1).

**Figure 9.**
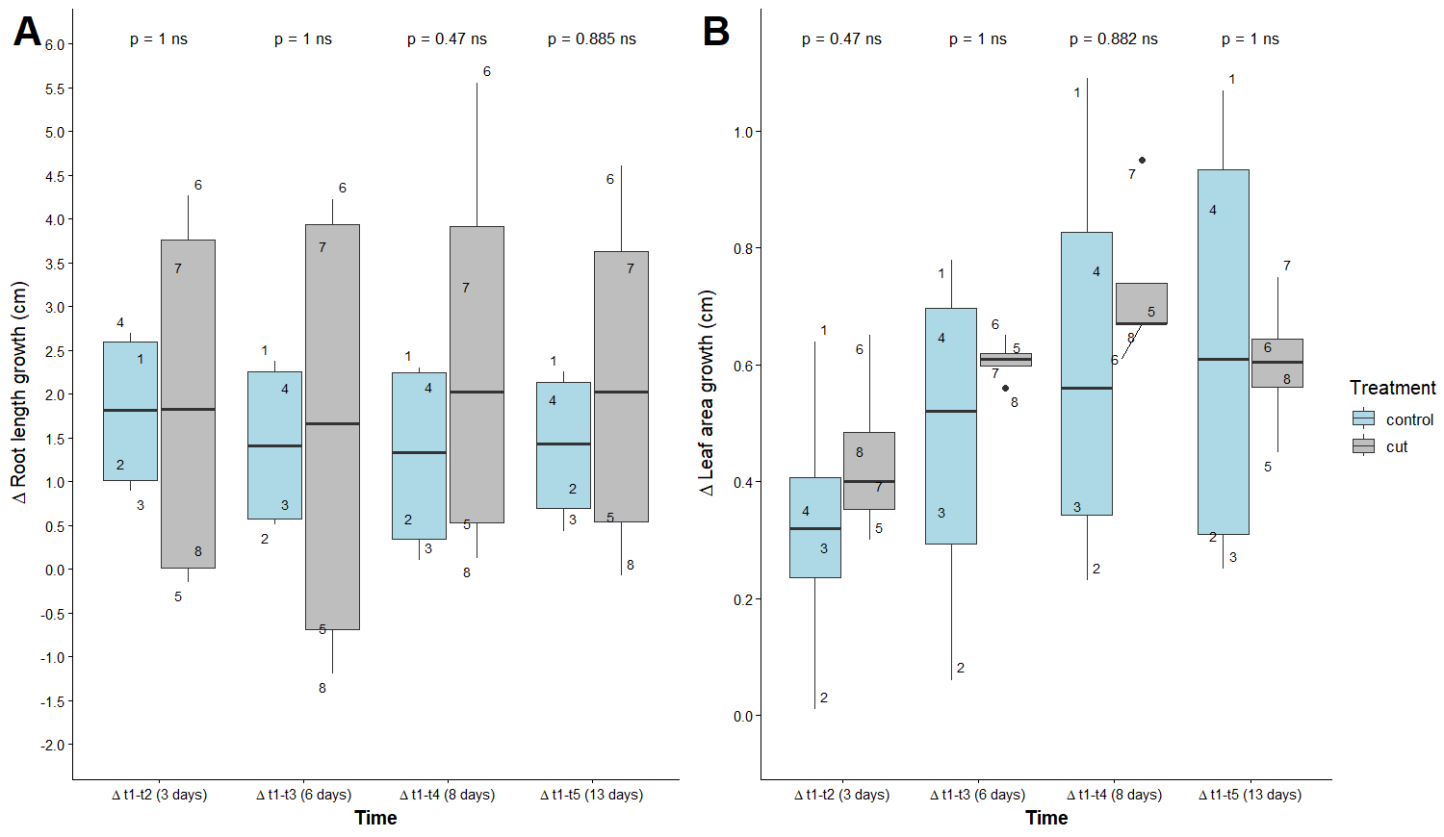
Difference in root length (A) and leaf area (B) between measurements of *Lactuca sativa* roots. Mann–Whitney test results are displayed above the boxplots; measurements over four time intervals (t2–t1, t3–t1, t4–t1, and t5–t1) with eight replicates per treatment, where each replicate corresponds to a single plant. Each point is a number indicating a single plant. ns, non-significant.

We found that control and cut treatments had no effect on root length or leaf area (Figure 9 and Table S1). Notable differences were found in the variation of data, where root length was more variable in cut treatments and with time compared to the control (p-value < 0.01; Figure 9A). The opposite pattern occurred for leaf area, where more variation was observed for control treatments than cut treatments over time (p-value < 0.01; Figure 9B). A total of 60% of the variation in root length could be explained by treatment, but only 19% was explained by leaf area (Figure 9 and Table S2).

**Figure 10.**
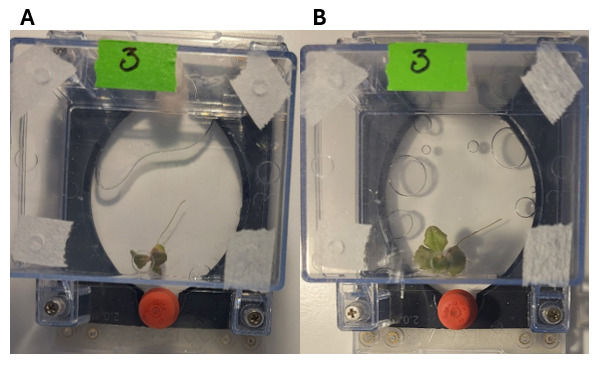
Type of images used to calculate differences in leaf area and root length between measurements of *Lactuca sativa* plant traits 4 days (A) and 14 days (B) after setup in EcoFAB. Other intervals were also measured but are not shown here.

We concluded that the control condition (only sterilization) gives the best germination, from a qualitative and quantitative point of view (Figures 6–9). We also recommend starting with a larger sample number to ensure plants can establish in EcoFABs (examples shown in Figure 10).

**C. *Fragaria vesca* “Hawaii 4”**

We have tested ten treatments to improve homogeneity and rate of germination for *Fragaria vesca*:

1. Control (only sterilization).

2. Scarification: Put around 20, sterilized 0.5 mm borosilicate beads in the Eppendorf tube containing seeds, shake for 30 s with the vortex to allow small abrasions on the seed coat*.

3. Heat: Put the Eppendorf tube containing the seeds in a water bath at 60 °C for 3 min.

4. Hydration/dry: Put seeds in MilliQ water for 24 h. Transfer the seeds to a Petri dish, cover them with Kimtech wipes and place the lid on the Petri dish. Place the dish into a desiccator for 24 h. Freeze the seeds in a cold room, so they are ready for use, similar to the non-treated seeds. These seeds can then be sterilized.

5. HCl for 30 s: Place seeds in 0.1 M HCl for 30 s.

6. HCl for 2 min: Place seeds in 0.1 M HCl for 2 min.

7. HCl for 30 s and scarification: combine processes.

8. HCl for 2 min and scarification: combine processes.

9. HCl, heat, and scarification: combine processes.

10. Hydration/dry and beads: combine processes.

*See Problem 1 in Troubleshooting.

We put 6 seeds in each Petri dish and determined the germination rate across time for every treatment. Similar to *Lactuca sativa*, we rated treatments by both germination rate and germination score (examples of scores for plant condition can be viewed in Figure 7).

**Figure 11.**
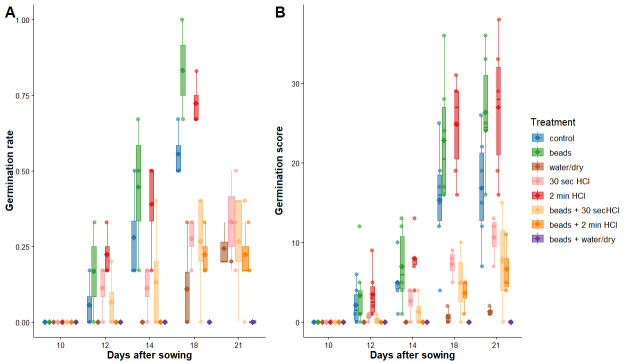
Germination rate (A) and score (B) across several seed priming treatments on *Fragaria vesca*. Three Petri dishes per treatment, six seeds each.

We found that three treatments tended to have higher germination rates: beads, HCl, and control (Figure 11A and Table S1). Whilst scarifying seeds with beads appears to be the best treatment, there was a greater variation in germination rate between Petri dishes (p-value < 0.01; Figure 11A and Table S2). Interestingly, only 15% of the variation in germination rate could be explained by treatment, whilst 45% was explained by time left in the Petri dish (Table S2). Germination score also tended to be higher in these three treatments (Figure 11B and Table S1). A larger proportion of variation in the data was explained by treatment (36%) and time left in the Petri dish (48%). Here, HCl and bead treatments were more variable in germination score by Petri dish (p-value < 0.01; Figure 11B and Table S2).

**Figure 12.**
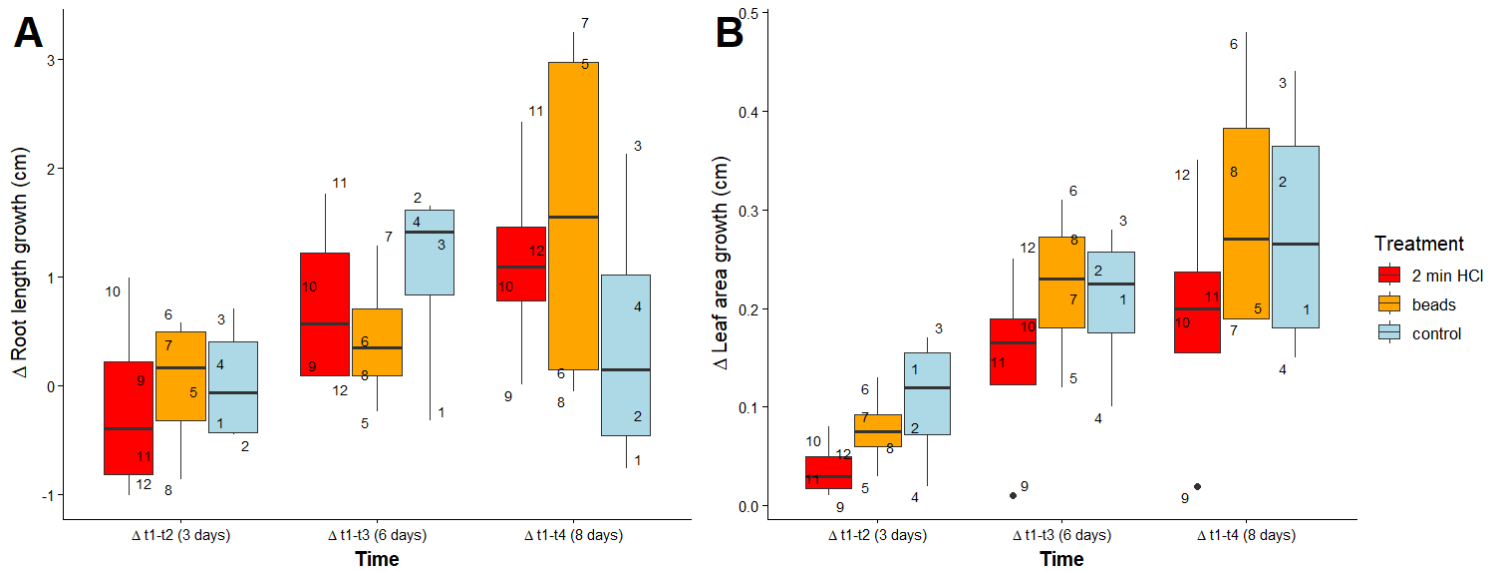
Difference in root length (A) and leaf area (B) between measurements of *Fragaria vesca*. Each point is a number indicating individual plants. Pairwise statistical comparisons using the Mann–Whitney test are presented in Table S1.

To determine if there were differences in plant traits by seed treatment, we transferred plants from beads, 2 min HCl, and control treatments to EcoFABs. No significant differences were observed in plant traits by treatment (Figure 12 and Table S1). The difference in root length increased significantly the longer plants were left in EcoFABs, explaining 20% of the variation (p-value = 0.03; Figure 12A and Table S2).

**Figure 13.**
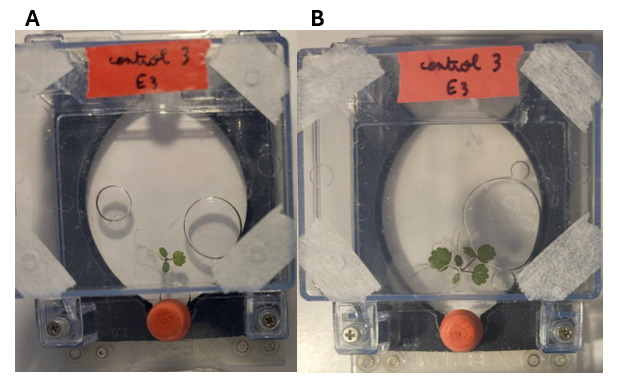
Type of images used for the calculation of differences in leaf area and length between measurements of *Fragaria vesca* plant traits 4 (A) and 14 days (B) after setup in EcoFAB. Other intervals were also measured but are not shown here.

In *F. vesca*, contamination during germination became a problem. As such, we increased the concentration of hypochlorite acid to 3% during the sterilization step (see Problem 1 in Troubleshooting). We then had increased success and growth in EcoFABs (examples shown in Figure 13). As such, for *F. vesca*, we recommend either the 2-min HCl seed treatment or scarification with beads for homogeneous plant traits in EcoFABs.

## General notes and troubleshooting

**General notes**

1. Opening the EcoFAB during setup steps is a repetitive task. To minimize these effects, we used an electronic screwdriver. Further steps may be required to comply with relevant operational health and safety standards.

2. When filling EcoFAB, move slowly and tilt the EcoFAB by raising the opposite port by 1 cm. If the EcoFAB leaks during this process, carefully tighten the screws at the base of the EcoFAB.

3. Note that all media is usually depleted of nutrients after 7 days, so it needs to be removed and replaced at least every 7 days.

**Troubleshooting**

**Problem 1:** We noticed contamination in a Petri dish with lettuce.

Possible cause: Borosilicate beads used for scarification do not stay sterilized between experiments.

Solution: Autoclave the beads the day before the treatment and sterilization of seeds.

**Problem 2:** We observed more fungal contamination in Petri dishes during strawberry germination.

Possible cause: Sterilization of seeds did not adequately remove microorganisms from the seed coat.

Solution: Increase bleach concentration to achieve stronger sterilization of the seeds.

## Supplementary information

The following supporting information can be downloaded here:

1. Table S1. Mann–Whitney results for differences between seed treatments for *Arabidopsis thaliana, Lactuca sativa*, and *Fragaria vesca*. P-values in bold indicate significance (p < 0.05) across measurements.

2. Table S2. Levene tests for the difference in variation across measurements for *Arabidopsis thaliana, Lactuca sativa*, and *Fragaria vesca* by group. Significance is indicated by p < 0.05 (in bold). Df1 corresponds to the degrees of freedom of the groups. Df2 corresponds to the residual degrees of freedom (total number of observations minus total number of groups). F value was used to test the equality of variances, and η^2^ indicates the proportion of variance explained (effect size).
